# Prévalence et facteurs associés aux candidoses vulvovaginales chez les femmes admises en consultation à l’Hôpital de Zone de Mènontin (Bénin)

**DOI:** 10.11604/pamj.2022.42.215.28984

**Published:** 2022-07-19

**Authors:** Brice Armand Fanou, Jean-Robert Klotoe, Victorien Dougnon, Amamath Monteiro, Charles Hornel Koudokpon, Frédéric Loko

**Affiliations:** 1 Unité de Recherche en Microbiologie Appliquée et Pharmacologie des Substances Naturelles (URMAPha), Laboratoire de Recherche en Biologie Appliquée (LARBA), Ecole Polytechnique d’Abomey-Calavi (EPAC), Université d’Abomey-Calavi, Cotonou, Bénin,; 2 Ecole Normale Supérieure de Natitingou, Université Nationale des Sciences, Technologie, Ingénierie et Mathématiques, Natitingou, Bénin,; 3 Hôpital de Mènontin, Ministère de la Santé, Mènontin, Bénin

**Keywords:** Candidose vulvovaginale, prévalence, facteurs associés, Bénin, Vulvovaginal candidiasis, prevalence, associated factors, Bénin

## Abstract

**Introduction:**

les candidoses vulvovaginales (CVV) sont des affections cosmopolites, très fréquentes et très récidivantes, liées à la rupture de l´équilibre vaginal et du mécanisme de l´immunité locale. L´objectif de cette étude est de déterminer la prévalence et les facteurs associés aux CVV chez les femmes admises en consultation à l´hôpital de Mènontin.

**Méthodes:**

il s´est agi d´une étude transversale à visée descriptive et analytique menée au Service de Gynéco-obstétrique de mars à août 2020. Les données sociodémographiques et médico-gynécologiques ont été collectées chez 1336 sujets. L´identification des espèces de Candida s´est basée sur les tests microbiologiques conventionnels.

**Résultats:**

la prévalence des candidoses est de 56,25% chez les femmes présentant une leucorrhée. Elle n´est pas associée aux facteurs sociodémographiques tels que la tranche d´âge, au statut matrimonial et à l´IMC. Au niveau de facteurs gynécologiques, l´état gestationnel; la couleur du col de l´utérus ainsi que la quantité et la consistance des leucorrhées sont en lien avec la survenue des candidoses. Les espèces les plus fréquentes sont Candida dubliniensis (36,11%), Candida albicans (29,17%).

**Conclusion:**

il existe huit espèces de Candida responsable des CVV au sud du Bénin. La connaissance des facteurs associés permettra la mise en place des stratégies de lutte appropriées de ces affections.

## Abstract (en)

**Introduction:**

vulvovaginal candidiasis (VVC) are cosmopolitan affections, very frequent and recurrent, condition due to disruption in vaginal balance and local immunity system. The purpose of this study is to determine the prevalence and factors associated with VVCs in women admitted to the Mènontin Hospital.

**Methods:**

we conducted a cross-sectional, descriptive and analytical study in the Department of Obstetrics and Gynaecology over the period March - August 2020. Sociodemographic, medical and gynecological data were collected from the medical records of 1336 subjects. Candida species were identified by conventional microbiological tests.

**Results:**

the prevalence of candidiasis was 56.25% in women with leucorrhea. It was not associated with sociodemographic factors such as age, marital status and BMI. Gestational age; the colour of the cervix and the amount and consistency of leucorrhoea were related to the occurrence of candidiasis. The most common species included Candida dubliniensis (36.11%) and Candida albicans (29.17%).

**Conclusion:**

eight species of candida are responsible for VVC in southern Benin. Knowing the associated factors allows for the implementation of appropriate control strategies.

## Introduction

Les candidoses, encore appelées monilioses [1,2] constituent des affections fongiques les plus fréquentes en pathologie humaine. Leur prévalence en Afrique tropicale varie entre 33% et 47% des infections opportunistes [3]. Parmi elles, on retrouve la candidose vulvovaginale (CVV) qui affecte le tractus génital féminin. Il s´agit d´une infection cosmopolite et très fréquente [4], représentant environ un tiers des cas de vaginite [5]. Elle est située au second rang des infections vaginales après la vaginose bactérienne [6-8]. Très récidivante, elle touche entre 138 et 140 millions de femmes dans le monde chaque année [7,9,10] où, 70-75% d´entre elles souffrent d´au moins un épisode de candidose vulvovaginale par an au cours de leur vie [4,11,12]. Leur incidence, ces 20 dernières années, a été multipliée par 2,5, contrairement aux vaginites à gonocoque et à *Trichomonas* qui ont parallèlement connu une réduction [13]. Cette maladie infectieuse liée à la rupture de l´équilibre vaginal et du mécanisme de l´immunité locale est alors favorisée par la colonisation vaginale par les espèces du genre *Candida* [4,14]. *C. albicans* demeure l'espèce la plus fréquente (77-95%) suivie des *Candida non albicans* (20-30%) dont la plus isolée est *C. glabrata* [15]. La nuisance de ces agents infectieux est généralement facilitée par différents facteurs de risque, et se manifeste par des leucorrhées, des prurits, un état d´inconfort vaginal, des difficultés dans les relations intimes qui exigent une prise en charge urgente [16].

La prévalence de la CVV varie d´un pays à un autre. Elle était de 35,52% sur une population de trois cent quatre-vingt-dix-sept (397) femmes à Yaoundé selon Keita *et al*. (2015) [17] et de 32,6% sur mille cent quarante et un (1141) patientes au Sénégal [18]. Au Bénin, très peu de données existent sur l´affection. Récemment, les travaux de Ogouyèmi-Hounto *et al*., (2014) [19] ont permis de savoir qu´à l´hôpital de la Mère et de l´Enfant, l´incidence des CVV était de 38,9% dans une population de cent trente et un (131) femmes, mais aucune information n´existe ni sur la virulence des souches circulantes, ni sur les facteurs associés. C´est pour répondre à cette insuffisance que la présente étude a été initiée chez les femmes admises en consultation dans le service de gynéco-obstétrique de l´Hôpital de Zone de Mènontin. Elle a pour objectifs de (i) déterminer la prévalence des CVV chez ces dernières; (ii) rechercher les facteurs associés à la survenue de CVV et (iii) identifier les espèces de Candida responsables.

## Méthodes

**Type d´étude:** il s´est agi d´une étude transversale à visée descriptive et analytique menée sous forme d´entretien couplée à la détermination de paramètres microbiologiques.

**Cadre d´étude:** l’étude s´est déroulée au service de gynéco-obstétrique de l´Hôpital de Zone de Mènontin, dans la ville de Cotonou au Bénin.

**Participants:** l´étude a porté sur toutes les femmes reçues en consultation au service de gynéco-obstétrique de l´Hôpital de Zone de Mènontin de mars à août 2020 et ayant données leurs consentements écrits pour participer à l´étude.

### Collecte des données

**Variables sociodémographiques et médico-gynécologiques:** l´entretien et l´examen gynécologique réalisé sur chaque patiente a permis de collecter des variables sociodémographiques (âge, statut matrimonial, nombre de partenaires sexuels, occupation) et médico-gynécologiques (état gestationnel, avortement, antécédents de santé, fréquence de douches vaginales, fréquence de change, aspect du col de l´utérus, aspect des leucorrhées). A l´issu de cette phase, des prélèvements de secrétions cervico-vaginaux (PCV) ont été effectués chez les femmes ayant présenté une leucorrhée en vue des examens microbiologiques.

**Identification des souches:** les analyses microbiologiques des PCV ont été réalisées à l´Unité de Recherche en Microbiologie Appliquée et Pharmacologie des substances naturelles (URMAPha) du Laboratoire de Recherche en Biologie Appliquée (LARBA) de l´Université d´Abomey-Calavi (UAC). Les souches ont été isolées puis identifiées sur la base des méthodes microbiologiques conventionnelles. En effet, chaque souche de *Candida* a été identifiée sur la base de données microscopiques, d´examens caractéristiques de la culture, formation du tube germinatif, fermentation des sucres, assimilation des sucres, aspect sur gélose CHROMagar, et sur gélose au tétrazolium (Tétrazolium reduction medium TRM) selon Khan *et al*.; Pandey *et al*., (2018) [20,21].

**Analyses statistiques:** les données ont été traitées et analysées avec le logiciel SPSS 22.0. Les variables qualitatives ont été exprimées sous forme de fréquence et les variables quantitatives sous forme de moyenne. La prévalence des CVV chez les femmes reçues en consultation gynécologique et présentant une leucorrhée a été déterminée par la formule:


$$P(\%)=\frac{Nombre\text{ de femme ayant une culture de PVC positive }à\text{ Candida}}{Nombre\text{ total ayant pr}ésentéuneleucorrhée}\times 100$$

Le test de Chi2 a été utilisé pour la comparaison des proportions de femmes atteintes de CVV pour chaque variable qualitative considérée (tranche d´âge, statut matrimonial, nombre de partenaires sexuels, occupation, état physiologique, avortement, antécédents de santé, fréquence de douche vaginales, fréquence de change, aspect du col de l´utérus, aspect - couleur - consistance des leucorrhées). La recherche des facteurs associés à la survenue de CVV a été faite par la régression logistique binaire de la variable dépendante (présence ou non de CVV) sur les autres variables. Le seuil de significativité a été fixé à 5% avec un intervalle de confiance de 95% pour les Obbs Ratio.

## Résultats

### Caractéristiques de l´échantillon des femmes présentant une leucorrhée

L´étude a porté 1336 femmes admises en consultation gynécologiques à l´hôpital de Mènontin parmi lesquelles cent vingt-huit (128) présentaient une leucorrhée soit 9,58%. Elles ont entre 15 et 58 ans avec une moyenne d´âge de 28 ans ± 7 ans. La tranche d´âge de 20 à 40 ans a été celle la plus représentée (76,56%) (Tableau 1). Plus de 75% de ces femmes vivaient en couple et 40% étaient en état de grossesse. Une (01) femme sur dix (10) était hypertendue. En ce qui concerne l´occupation, le nombre de femmes salariées prédominaient, mais ne diffèrent pas statistiquement de celles qui ne sont pas dans un emploi rémunéré. Plus de 95% d´entre elles déclarent avoir un seul partenaire sexuel (Tableau 1).

**Tableau 1:** répartition des données sociodémographiques

Paramètres		Fréquences	Proportions (%)	p-value
Tranches d´âge	**< 20 ans**	23	17,97	**0,000**
	**[20 à 40[**	98	76,56	
	**> 40ans**	7	5,47	
Indice de masse corporelle (IMC)	**Poids normal**	49	38,28	**0,307**
	**Surpoids**	44	34,38	
	**Obésité**	35	27,34	
Situation matrimoniale	**Célibataire**	28	21,88	**0,000**
	**Mariée**	100	78,13	
Nombre de partenaires	**un**	123	96,09	**0,000**
	**Plus d´un**	5	3,91	
Occupation	**Employée**	73	57,03	**0,133**
	**Non employé**	55	42,97	
Etat physiologique	**Femme non enceinte**	77	60,16	**0,027**
	**Femme enceinte**	51	39,84	
Termes de grossesse	**1er trimestre**	14	27,45	**0,461**
	**2e trimestre**	19	37,25	
	**3e trimestre**	18	35,29	
Antécédent HTA	**Non hypertendue**	115	89,84	**0,000**
	**Hypertendue**	13	10,16	

### Prévalence de candidoses et influence de variables de l´étude

La prévalence de la candidose vulvo-vaginale chez les femmes présentant une leucorrhée a été de 56,25%. La CVV touche toutes les tranches d´âge. Cependant, les femmes de moins de 20 ans ont le taux le plus élevé, 65% contre 28,57% pour celles de plus de 40 ans. Toutefois, cette différence n´est pas statistiquement significative (p=0,231). Les autres variables sociodémographiques: situation matrimoniale; le type d´emploi; le nombre de partenaires n´ont montré aucun lien avec le taux de CVV (Tableau 2).

**Tableau 2:** comparaison des proportions de candidose vulvo-vaginale suivant les variables de l´étude

Variables		Candidose		Total	Fréquence de CCV	p-Value
		Absence	Présence			
**Tranche d´âge**	< 20 ans	8	15	23	65,22%	0,231
	[20 à 40[	43	55	98	56,12%	
	> 40 ans	5	2	7	28,57%	
**Situation matrimoniale**	Célibataire	12	16	28	57,14%	0,545
	Mariée	44	56	100	56,00%	
**Nombre de partenaires**	un	54	69	123	56,10%	0,618
	plusieurs	2	3	5	60,00%	
**Emploi**	Non salarié	20	35	55	63,64%	0,100
	Salarié	36	37	73	50,68%	
**Indice de masse corporel**	Poids normal	18	31	49	63,27%	0,420
	Surpoids	22	22	44	50,00%	
	Obésité	16	19	35	54,29%	
**Hypertension**	Non	51	64	115	55,65%	0,460
	Oui	5	8	13	61,54%	
**Grossesse**	Non	40	37	77	48,05%	0,022
	Oui	16	35	51	68,63%	
**Trimestre de grossesse**	1er trimestre	5	9	14	64,29%	0,227
	2e trimestre	5	14	19	73,68%	
	3e trimestre	6	12	18	66,67%	
**Aspect du col de l’utérus**	Rouge	43	66	109	60,55%	0,018
	Normal	13	6	19	31,58%	
**Couleur de la leucorrhée**	Blanchâtre	41	50	91	54,95%	0,641
	Jaunâtre	15	22	37	59,46%	
**Quantité de la leucorrhée**	Infirme	12	11	23	47,83%	0,001
	Peu abondant	31	21	52	40,38%	
	Abondant	13	40	53	75,47%	
**Consistance leucorrhée**	Glaireuse	4	2	6	33,33%	0,026
	Laiteuse	37	35	72	48,61%	
	Cailleuse	8	27	35	77,14%	
	Pâteux	7	8	15	53,33%	
**Nombre de douches / jour**	2	39	43	82	52,44%	0,474
	plus de 2	16	28	44	63,64%	
**Fréquence de change**	Rarement	9	17	26	65,38%	0,304
	Souvent	34	34	68	50,00%	
	Très souvent	13	21	34	61,76%	
Total		56	72	128	56,25%	

Au niveau des variables médicales, il faut noter que ni l´hypertension ni l´indice de masse corporelle (IMC) n´influençait significativement l´incidence de la CVV. Toutefois, le taux de CVV est plus élevé chez les femmes hypertendues 61,54% par rapport à celles qui ne le sont pas 55,65% (Tableau 2). Dans cette étude, les femmes enceintes ont été statistiquement plus touchées (p = 0,022) que les non enceintes par la CVV (68,63% contre 48,05% respectivement). Toutefois, l´âge gestationnel n´influence pas significativement la prévalence le taux de CVV même si elle était plus élevée aux deuxième trimestre (Tableau 2). En ce qui concerne les paramètres gynécologiques, la présente étude montre que plus de la moitié des femmes consultant pour la présence de leucorrhée souffre de la CVV. Il existe non seulement un lien étroit entre la CVV et la présence de leucorrhée, mais on remarque également une augmentation significative du taux de CVV lorsqu´elles deviennent abondantes (abondante 75,47% contre infime 47,83% avec p = 0,001) et lorsque leur consistance change (laiteuse 48,61%; cailleuse 77,14% et pâteuse 53,33% contre glaireux: 33,33 avec p = 0,026). Aussi, on note un lien entre l´aspect du col de l´utérus et le taux de CVV chez les femmes. Ici, le taux de CVV est statistiquement plus élevé chez les femmes présentant un col de l´utérus rouge (Tableau 2).

### Facteurs associés à la survenue de CVV

Le Tableau 3 présente le résultat de l´analyse multivariée par la régression logistique binaire. Il permet d´identifier quatre facteurs associés à la survenue de CVV. En effet, le facteur grossesse augmente de plus de deux le risque de développer une CVV (OR 2,612). Les autres facteurs associés ont rapport avec l´aspect du col de l´utérus et la leucorrhée. Avec un OR de 2,835 (p=0,037) on remarque lorsque le col de l´utérus passe de son aspect normal à une couleur rouge, le risque pour la femme d´être atteinte d´une CVV est triplet. Quant à la leucorrhée, son abondance augmente le risque de 2 à 6 fois. Et lorsque cette leucorrhée a un aspect cailleux le risque est 9 fois plus élevé (Tableau 3).

**Tableau 3:** répartition des femmes atteintes de la CVV en fonction des facteurs gynécologiques

Variables			C 95% pour OR		
		OR	Inférieur	Supérieur	p-value
**Grossesse**	Non	1			
	Oui	2,612	1,057	6,450	0,037
**Aspect du col de l´utérus**	Normal	1			
	Rouge	2,835	1,485	9,592	0,039
**Quantité de la leucorrhée**	Infirme	1			
	Peu abondant	1,856	1,122	4,865	0,031
	Abondant	2,617	1,875	6,562	0,014
**Consistance de la leucorrhée**	Glaireuse	1			
	Laiteuse	1,023	0,486	1,623	0,100
	Pâteux	2,925	1,296	4,781	0,025
	Cailleuse	8,555	4,236	15,781	0,012

### Espèces de *Candida* identifiées

**Figure 1:**
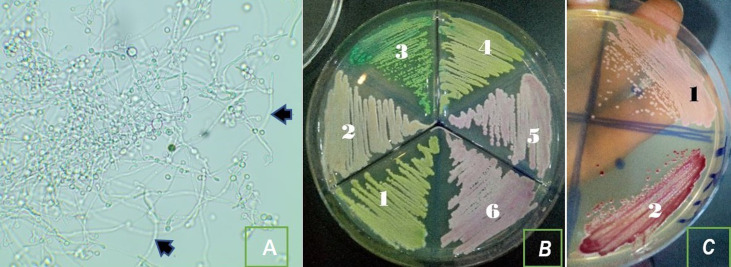
aspects de quelques levures du genre Candida observées au microscope X40

**Figure 2:**
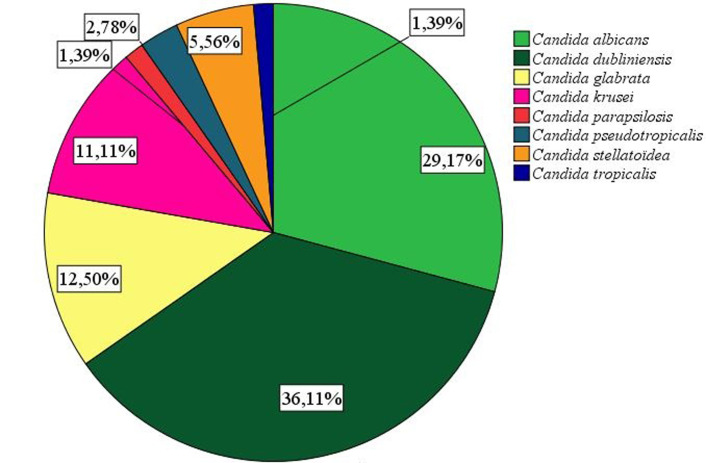
distribution des espèces de Candida identifiées

Soixante-douze (72) espèces de Candida (C.) responsables des CVV ont été isolées et identifiées phénotypiquement (Figure 1). Sur ces 72 isolats, les espèces non-albicans (n= 51) étaient prédominantes, représentant 70,83% (Figure 2). *Candida dubliniensis* (36,11%), *Candida albicans* (29,17%), *Candida glabrata* (12,50%) et *Candida krusei* (11,11%) étaient les espèces les plus isolées (p = 0,000). D´autres espèces telles que *C. stellatoïdea, C. pseudotropicalis, C. parapsilosis* et *C. tropicalis* ont été également identifiées responsables des CVV.

## Discussion

La candidose vulvovaginale, l´une des infections féminines la plus répandue à travers toute la planète, très récidivante et ainsi, constituent un problème de santé publique majeur dans la vie des femmes présentant une plainte de pertes vaginales anormales [22,23]. Elle représente l'une des causes les plus fréquentes d'écoulement, d'infection vaginale et de consultations gynécologiques des femmes [24]. Cette étude a porté sur 1336 femmes admises en consultation gynécologique pour diverses raisons. Parmi elles, 128 (soit 9,58%) d´âge compris entre 15 et 58 ans et présentant des manifestations physiques d´une altération de la flore vaginale ont fait objet d´étude approfondie.

Dans notre étude, le diagnostic biologique de la candidose vulvo-vaginale a été confirmé chez soixante-douze (72) des femmes présentant des leucorrhées soit une prévalence de 56,25%. Cette prévalence est largement supérieure à celle obtenue par Ogouyèmi-Hounto *et al*. [19] qui était de 36,9% cinq ans plus tôt à l´Hôpital de la Mère et de l´Enfant de la Lagune (HOMEL) au Bénin. La prévalence de la CVV varie en fonction de l´aire géographique où l´étude a été effectuée, en fonction des cibles ou même tributaire de la taille d´échantillon ou de la période de l´étude (climat) [25]. L´incidence de cette affection étant en hausse constante [13], cela pourrait d´une part justifier l´écart observé.

D´autre part, cette différence dans les taux obtenus pourrait également être liée au fait que dans la présente étude, le diagnostic était systématique chez toutes les femmes admises au service gynécologique de l´Hôpital de Mènontin et présentant des leucorrhées alors que celle de Ogouyemi s´était intéressée uniquement aux femmes auxquelles étaient demandé un examen cervico-vaginal. Par ailleurs, d´autres études ont montré que plus d´une femme sur deux étaient atteintes de CVV. En effet, ElFery *et al*. avaient obtenu dans leur étude 50,4% de cas de CVV en Egypte [26] et une prévalence de 55,4% obtenue dans une étude au Cameroun depuis déjà une vingtaine d´années, proche de la nôtre avait été rapporté par Khan *et al*. en 2018 [20]. De plus, selon certains auteurs, les prévalences obtenues par le laboratoire d´analyses médicales seraient sous-estimées puisque les recherches ne s´intéressent souvent qu´au cas de vaginites qui s´affichent [20]. Ainsi, en Afrique et ailleurs, plusieurs auteurs ont récemment obtenu des prévalences plus basses variant entre 25 et 50% [6,8,15,16,20,27,28].

Dans cette étude, nous avions noté des variations du taux de CVV en fonction de certains paramètres sociodémographique. L´âge moyen des femmes était de 28 ± 7,074 ans et celles appartenant à la tranche d´âge compris entre [21-33] prédominaient avec une proportion de 71,87%. La prévalence de la candidose dans cette tranche d´âge reflétait la prévalence générale de 56,25%. D´une part, cet intervalle d´âge correspond bien à celui où, les imprégnations hormonales sont au maximum de leurs activités et d´autre part à celui où les couples, le plus souvent en quête d´enfants, sont sexuellement actifs. Il s´agit donc par excellence de la tranche d´âge où les femmes sont plus exposées aux vaginites dont la candidose [5]. Nos observations en ce qui concerne la tranche d´âge prédominante corroborent celles d´autres travaux en Afrique où la tranche d´âge la plus représentée était celle de 20-35 ans [8,29]. Ces mêmes observations ont été faites dans d´autres travaux en dehors de l´Afrique [20,23]. Mtibaa *et al*., dans une étude s´intéressant à l´étiologique de la CVV avaient trouvé que la prévalence de la candidose était aussi élevée dans cette tranche d´âge [16]. Néanmoins, la tranche d´âge des moins de 20 ans affichait statistiquement une prévalence plus élevée que la tranche prédominante (65,22% contre 56,16% respectivement) significativement moins représentée. Cet constat avait été également fait dans une récente étude rétrospective au Sud de la Pologne sur les infections vaginales où les auteurs avaient montré que les candidoses prédominaient chez les femmes avec 32,3% et 25% respectivement dans les tranches d´âge de 15 à 24 ans et de 25 à 35 ans [30]. Il s´agit en fait des tranches d´âge correspondant à la pleine période d´activité sexuelle faisant ainsi penser la possibilité d´une transmission de *Candida* par voie sexuelle [19].

Le statut matrimonial des femmes, leurs nombres de partenaires sexuels et leurs occupations n´avaient pas une influence significative sur le taux des CVV. Nos résultats sont en phase avec ceux obtenus dans quelques études sur les facteurs associés à la CVV au Ghana et au Cameroun. En effet les auteurs de ces travaux n´avaient observé aucune différence significative entre les femmes mariées et les célibataires même si ces études révélaient que les premières prédominaient leur échantillon [6,25,29]. A l´opposé, les travaux effectués en Côte d´Ivoire et au Sénégal avaient montré l´existence d´un lien entre la prévalence des CVV et statut matrimonial, mais leurs prévalences n´atteignaient pas 50% [8,22]. S´intéressant au nombre de partenaires sexuels, Bitew & Abebaw, et Brandolt *et al.*, au Brésil avaient observé, qu´il existait une différence significative entre les proportions des femmes touchées par la CVV [6,31] contrairement à Mogtomo *et al.*, pour qui ces prévalences ne variaient pas significativement [29]. Au niveau de la fréquence de douches vaginales, nos observations concordent avec celles de Konadu *et al*. qui avaient cependant obtenu une proportion moindre de 36,2% [25]. Trois femmes sur cinq (57,03%) environ avaient une occupation. Konadu *et al*. avaient obtenu une proportion voisine (62,6%) de la nôtre [25] mais une proportion moindre (52%) était obtenue par [3^2^] avec 52% de non employées. Notre étude comme celui de Konadu *et al*. s´étant effectuée sur des populations urbaines contrairement à celui de Ekpenyoung, ces observations pourraient bien se justifier; la vie en milieu urbain exige quasiment à la femme un emploi afin de contribuer aux dépenses du foyer contrairement à la conception de la femme en milieu rural.

Cette étude sur les femmes admises au service de gynécologie de l´Hôpital de Mènontin a montré que l´abondance de la leucorrhée, leur état physiologique (enceinte ou non) influençaient significativement la prévalence de la CVV. Les symptômes les plus fréquents chez les femmes, motifs de leur visite en service de gynécologie étaient les leucorrhées (89,84%) puis le prurit vulvaire (10,16%). Il existait dans cette étude un lien étroit entre la présence des leucorrhées et la candidose. Selon Pizzorno *et al*. [33], le principal symptôme de la candidose est la démangeaison vulvaire (parfois grave) accompagné ou non de pertes épaisses (souvent fréquentes), cursives ou de “fromage blanc” qui adhèrent aux parois vaginales qui caractérisent généralement l´infection à levures, mais dont l’absence n'exclut pas la présence de *Candida*. D´une part, [34] avaient quasiment le même taux de leucorrhée (89,70%), très proches de ceux obtenus dans notre étude mais une prévalence du prurit plus marquée (77,28%) et avaient également conclu que d´autre part, la candidose et ces paramètres étaient fortement corrélés. Dans une récente étude, comme dans la présente où la majorité (85,16%) des femmes avaient leur col normal avec des pertes abondantes (82,03%), de couleur blanchâtre (71,09%) et homogènes (56,25%), Sylla *et al*. étaient parvenues à de résultats semblables avec des proportions 65,21% 66,08%, 77,91% et 59,95% respectivement pour l´aspect du col et les différents aspects des pertes [8].

Pour ce qui est de l´état physiologique, la prévalence de la CVV augmentait significativement chez les femmes enceintes (68,63%) par rapport aux femmes non enceintes (48,05%). Le lien étroit constaté entre l´incidence élevée de la candidose et l´état physiologique des femmes corroborent les résultats d´autres travaux qui se sont intéressés à ces cibles [16,23,3^1^]. L´état gestationnel favoriserait la survenue de candidose selon plusieurs auteurs [5,23,35,3^6^]. Ce constat se justifierait selon certains auteurs qui avaient conclu que la candidose augmenterait 10 à 20 fois plus pendant la grossesse en raison d'un pH vaginal élevé, d'une augmentation du glycogène épithélial vaginal, d'une glycémie élevée et d'une glycosurie intermittente [13,28]. De plus, l’incidence était plus élevée chez les femmes abordant les deuxième et troisième trimestres que chez celles au 1er trimestre de grossesse, mais sans différence significative. Konadu *et al*. (2019) avaient observé également que le terme de la grossesse n´influençait pas significativement l´incidence de la CVV chez les femmes enceintes.

Autrefois liée à *Candida albicans*, seule espèce considérée pathogène, plusieurs espèces sont de plus en plus impliquées dans les CVV ces dernières décennies [4,37-39]. A l´issue de cette étude, 29,17% de *C. albicans* et 69,83% de *Candida non albicans* avaient été identifiés. La prédominance des espèces non-*albicans* corroborent d´autres travaux qui avaient identifié dans ce même cadre plus de souches non-*albicans* que de *C. albicans* [15,28,40,4^1^]. Récemment, Ghaddar *et al*., dans une étude comparant la fréquence des espèces isolées des CVV selon l´aire géographique, faisaient remarquer que les souches non-*albicans* étaient plus impliquées dans les CVV en Afrique que sur d´autres continents [28]. Dans notre étude, l´espèce *C. dubliniensis* a été l´espèce prédominante suivie de *C. albicans*, de *C. glabrata, C. krusei*, de *C. stellatoïdea*, de *C. tropicalis*et *Candida parapsilosis*. Quelques auteurs avaient effectué les mêmes observations quant à la prédominance des espèces par les NAC mais issaient *C. glabrata* en tête de classement et *C. albicans* occupaient respectivement le troisième et le deuxième rang [16,4^1^]. Mais de nombreux travaux sur les CVV avaient obtenu une distribution contraire à la nôtre concernant la prédominance des NAC selon lesquels dans l´ordre, *C. albicans* était l´espèce largement prédominante suivie souvent de *C. glabrata*, de *C. krusei*, de *C. tropicalis*, de *C. parapsilosis* [4,6,20,24,25,28,30]. Selon ElFeky *et al*. [26], dans le monde entier, l´espèce *C. albicans* dans des cas de VVC était identifié entre 47% et 89%. La prédominance de *C. dubliniensis* (36,11%) dans notre étude était contraire à la littérature où la levure la plus isolée chez l´homme est *C. albicans* [36]. Cette espèce qui partage avec *C. albicans* la plupart des caractères morphologique avaient également été isolée par d´autres auteurs [20]. La discordance observée pourrait être due à la confusion morphologique que crée *C. dubliniensis* avec *C. albicans*; les deux espèces filamentant en milieu sérique à 37°C et présentant des caractères similaires sur les milieux usuels auxquels on a souvent recouru pour leur identification.

Ainsi, son identification dans la présente étude ajoutée à la divergence dans la distribution pourrait trouver son explication dans les méthodes d´identification utilisées. Dans notre étude, le milieu Sabouraud au TTZ a permis de différencier ces deux espèces. En revanche, on se rendrait bien compte que sans différentiation aucune, les proportions de ces deux espèces ensembles feraient seules la proportion de *C. albicans* et ainsi ferait de lui l´espèce prédominante comme dans la plupart des travaux. *C. krusei*, une espèce retrouvée le plus souvent dans les produits laitiers [42] fait partie des espèces majoritairement isolées de notre étude alors qu´elle l´était moins dans d´autres résultats d´autres auteurs. La présence de cette souche pourrait avoir une origine buccale. En effet, ces femmes pourraient avoir été contaminées par leurs partenaires sexuels par des pratiques sexuelles bucco-vaginales (le cunnilingus). Dans une étude récente, le spectre des espèces fréquemment identifiées responsables des CVV s´élargie avec une nouvelle *C. lusitaniae*, espèce rarement citée dans ce type d´infection mais qui est de plus en plus identifiée parmi les quatre espèces majoritaires responsables de la CVV [6,15]. Par ailleurs, les divergences dans la répartition des espèces liées à l´aire géographique où l´étude a été effectuée doivent être prises en compte parmi les facteurs épidémiologiques qui interviennent également dans l´infection de la muqueuse vaginale par des *Candida spp*.[28,3^1^].

## Conclusion

En somme, cette étude a montré que la CVV constitue une affection qui touche plus d´une femme sur deux dans la population et est fortement influencée par certains facteurs tels que l´état de grossesse, l´aspect du col de l´utérus et des caractéristiques de la leucorrhée. Elle est due à plusieurs espèces de Candida dominée par le complexe *C. albicans*/ *C. dubliniensis* suivies des espèces non-albicans dont les plus représentées sont: *C. glabrata, C. krusei* et *C. stellatoïdea*.

### Etat des connaissances sur le sujet

- Il est connu que la prévalence des candidoses vulvovaginales varie d´un pays à un autre;
- Elle était de 35,52% sur une population de trois cent quatre-vingt-dix-sept (397) femmes à Yaoundé selon Kechia et al. (2015) et de 32,6% sur mille cent quarante et un (1141) patientes au Sénégal (Khadime Sylla, 2018);
- Au Bénin, très peu de données existent sur l´affection, récemment, les travaux de Ogouyèmi-Hounto et al., (2014) ont permis de savoir qu´à l´hôpital de la Mère et de l´Enfant, l´incidence des CVV était de 38,9% dans une population de cent trente et un (131) femmes mais aucune information n´existe ni sur les types des souches circulantes, ni sur les facteurs associés.

### Contribution de notre étude à la connaissance

- La présente étude a permis, pour la première fois au Bénin d´identifier 6 autres espèces de candida en plus de deux (C. albicans et C. glabrata) indiqués auparavant comme responsable de CVV;
- Contrairement à ce qui était signalé jusqu´à présent dans la littérature scientifique donnant C. albicans comme principal responsable de CVV au Bénin, la présente étude montre que c´est plutôt C. dubliniensis qui est l´espèce prédominante en 2020; par ailleurs le présent travail a montré que l´aspect du col et les caractéristiques de la leucorrhée sont des facteurs associés aux CVV;
- Il permet aussi de savoir, par comparaison aux résultats de 2014, que la prévalence des CVV a une tendance en hausse au Bénin; les informations recueillis permettront de mieux comprendre les causes des candidoses vulvovaginales et les mesures de prévention à préconiser.

## Remerciements

Aux autorités de l´hôpital de Zone de Mènontin et aux femmes qui ont accepté de participer à l´étude.
